# Derivation of a Myeloid Cell-Binding Adenovirus for Gene Therapy of Inflammation

**DOI:** 10.1371/journal.pone.0037812

**Published:** 2012-05-18

**Authors:** Michael O. Alberti, Justin C. Roth, Mourad Ismail, Yuko Tsuruta, Edward Abraham, Larisa Pereboeva, Stanton L. Gerson, David T. Curiel

**Affiliations:** 1 Division of Human Gene Therapy, Departments of Medicine, Obstetrics and Gynecology, Pathology, Surgery, University of Alabama at Birmingham, Birmingham, Alabama, United States of America; 2 Gene Therapy Center, University of Alabama at Birmingham, Birmingham, Alabama, United States of America; 3 Division of Hematology and Oncology, Department of Medicine, Case Comprehensive Cancer Center, Case Western Reserve University, Cleveland, Ohio, United States of America; 4 National Center for Regenerative Medicine, Case Comprehensive Cancer Center, Case Western Reserve University, Cleveland, Ohio, United States of America; 5 Department of Medicine, University of Alabama at Birmingham, Birmingham, Alabama, United States of America; University of Chicago, United States of America

## Abstract

The gene therapy field is currently limited by the lack of vehicles that permit efficient gene delivery to specific cell or tissue subsets. Native viral vector tropisms offer a powerful platform for transgene delivery but remain nonspecific, requiring elevated viral doses to achieve efficacy. In order to improve upon these strategies, our group has focused on genetically engineering targeting domains into viral capsid proteins, particularly those based on adenovirus serotype 5 (Ad5). Our primary strategy is based on deletion of the fiber knob domain, to eliminate broad tissue specificity through the human coxsackie-and-adenovirus receptor (hCAR), with seamless incorporation of ligands to re-direct Ad tropism to cell types that express the cognate receptors. Previously, our group and others have demonstrated successful implementation of this strategy in order to specifically target Ad to a number of surface molecules expressed on immortalized cell lines. Here, we utilized phage biopanning to identify a myeloid cell-binding peptide (MBP), with the sequence WTLDRGY, and demonstrated that MBP can be successfully incorporated into a knob-deleted Ad5. The resulting virus, Ad.MBP, results in specific binding to primary myeloid cell types, as well as significantly higher transduction of these target populations *ex vivo*, compared to unmodified Ad5. These data are the first step in demonstrating Ad targeting to cell types associated with inflammatory disease.

## Introduction

Viral vectors are currently the most efficient means of gene transfer into a number of cell types. However, their use for gene-based therapies is often limited by the lack of vectors that can achieve specific and efficient gene transfer into target cells of interest. This is typically due to either broad tissue expression of viral receptors, which limit vector specificity, or a complete lack of receptor expression on the target cells [1]. Thus, much effort has gone into de-targeting viral vector tropism to ubiquitously-expressed receptors and retargeting them towards receptors that are unique to cell types associated with a particular pathology.

Adenovirus serotype 5 (Ad5)-based vectors are particularly suited for these de-targeting/retargeting efforts, as they have a well-characterized biology, and Ad capsid proteins are permissive to the significant alterations required for tropism changes [2]. Further, the functionally distinct two-step mechanisms by which Ad5 mediates cell attachment and entry facilitates targeting efforts. The knob domain of the Ad5 fiber protein mediates viral attachment via high-affinity binding to the human coxsackie-and-adenovirus receptor (hCAR) [3]. Subsequent to the attachment step, an Arg-Gly-Asp (RGD) motif in the penton base capsomer associates with cellular α_v_ integrins, triggering viral internalization and intracellular trafficking of the viral particle [4]. Thus, changes that redirect attachment to novel receptors are unlikely to perturb Ad internalization activity.

Different strategies have been employed to limit the broad tissue-specificity of Ad5 vectors, and redirect the viral tropism to particular cell types. Early studies described the use of bi-specific adapter proteins, composed of ligands fused to the ecto-domain of hCAR, to block Ad5-hCAR interactions and simultaneously target the virus to novel receptors [5]–[7]. These two-component systems provide an easy means to broaden the spectrum of targeting ligands that can be attached to Ad, particularly bulky moieties, such as single-chain antibodies (scFv), that often destabilize the capsid when incorporated directly into the virus [8], [9]. Further, scFv require routing through the endoplasmic reticulum for proper folding, but are redirected to the nucleus when fused to Ad capsid proteins [8]. Nevertheless, two-component systems are incompatible with oncolytic Ad or conditionally replicating Ad (CRAd) vectors, as the adapters are lost after the first round of infection. These systems are also difficult to optimize and require additional regulatory approvals for clinical translation.

Despite the utility of two-component targeting systems, a number of studies have continued to improve upon the conditions permissive for stabilized and efficient genetic incorporation of targeting ligands into the Ad capsid. Since specificity is provided at the attachment phase, optimal targeting strategies utilize insertion of ligand moieties within the fiber knob domain (reviewed in [1], [2]). Indeed, genetic insertion of targeting moieties into the HI-loop of the fiber knob domain has provided a successful platform for Ad retargeting strategies [10]–[12], but these insertions do not completely abrogate Ad tropism to hCAR, unless the hCAR-binding sites are also deleted [13], [14].

Knob-deleted vectors offer two significant advantages over their knob-insertion counterparts: First, knob has been shown to be significantly immunogenic [15]. Second, it has recently been demonstrated that soluble blood factors are responsible for Ad5 transduction of hepatocytes following intravascular injection. This effect is primarily mediated by factor X (FX) interactions with the Ad5 hexon protein [16], [17]; however, earlier reports also suggest a role for FIX/knob-mediated liver transduction *in vivo* [16], [18]. Thus, strategies that eliminate immunogenic domains, while still achieving significant targeting, offer improved clinical potential.

In this regard, our lab [19], [20] and others [21], [22] have evaluated knob-deleted targeting platforms. A number of proof-of-principle *in vitro* studies have highlighted the potential of this strategy [9], [19], [20], [23]. Recently, IL-13 incorporation into another knob-deleted platform was demonstrated to allow efficient and specific transduction of IL-13Rα2-expressing cells *in vivo* [24]. However, examples of knob-deleted platforms that target clinically relevant cell types are limiting. Further, additional targeting mechanisms will be required to route intravascularly-administered virus to extracellular sites of pathology.

Since a number of diseases are characterized by large numbers of inflammatory myeloid cell infiltrates [25], we sought to redirect Ad tropism to myeloid lineage leukocytes. In doing so, we hypothesize that systemically injected myeloid cell-targeted Ad may transduce or traffic with the cells and localize therapeutic gene expression to extravascular sites of pathology. Herein, we describe the use of phage biopanning to identify a myeloid cell-binding peptide (MBP) with the sequence WTLDRGY, and show successful incorporation of MBP into our knob-deleted fiber platform. The resulting virus, Ad.MBP, specifically binds and transduces myeloid cell types *ex vivo*. These studies demonstrate the feasibility of myeloid cell-targeting and are an important first-step for endpoints aimed at targeted gene therapy for inflammatory disease.

## Results

### Identification of a myeloid-binding peptide (MBP)

**Figure 1:**
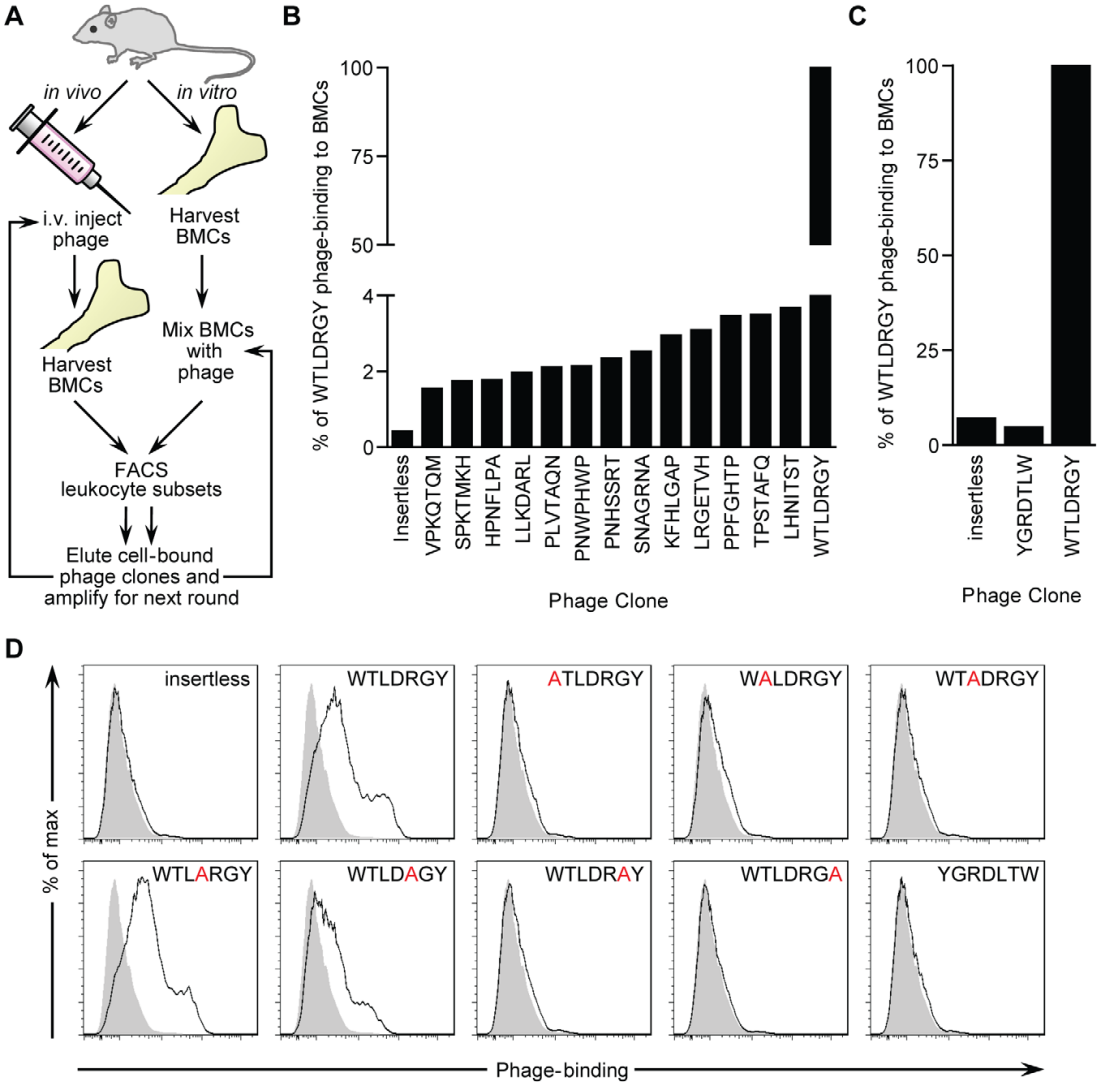
Identification of a myeloid cell-binding peptide (MBP) by phage display. (**A**) Schematic representation of the *in vitro* and *in vivo* panning strategies used to identify putative myeloid-binding phage peptides from a cysteine-constrained random heptapeptide M13 phage display library. A complete list of isolated phage peptides is presented in **Table 1**. (**B**) Flow cytometric detection of binding of individual biotinylated-phage clones to bone marrow cells (BMCs) at 4°C. Background binding was assessed with insertless control phage. (**C**) Flow cytometric detection of binding of WTLDRGY and YGRDLTW phage clones to BMCs at 4°C. Background binding was assessed with insertless control phage. (**D**) Phage binding to CD11b^+^ BMCs following scanning alanine mutagenesis of the WTLDRGY sequence. Each residue of the WTLDRGY sequence was substituted with an alanine (A) and cloned into the original M13 phage display platform. Phage clones were then amplified and binding was assessed as in **B** and **C**. Gray histograms indicate no phage control staining. Representative experiments are shown for **B–D**.

Phage display biopanning is a technique that has been successfully used to identify ligands that bind to specific targets *in vitro* as well as *in vivo* [26], [27]. In order to identify novel myeloid cell-targeting peptides we employed two complementary phage biopanning strategies (**Fig. 1A**). First, a loop-constrained random heptapeptide phage display library was panned over murine bone marrow cells (BMCs) *in vitro*. Leukocytes were isolated by fluorescence-activated cell sorting (FACS) and cell-bound phage were eluted, amplified, and used as input for subsequent pans. A single phage peptide, with the sequence WTLDRGY, was identified after three rounds of *in vitro* panning (**Table 1**). For the second approach, the phage library was infused into the tail vein of one mouse. Shortly after injection, BMCs were extracted and leukocytes were isolated by FACS. Leukocyte-bound phage were eluted, amplified and used as input for subsequent pans in additional mice. Three rounds of *in vivo* panning produced 15 individual phage peptides (from 32 clones sequenced) but failed to generate a consensus sequence (**Table 1**). However, the WTLDRGY phage peptide identified *in vitro* was also identified in multiple rounds of *in vivo* panning.

**Table 1:** Phage peptide candidate list.

Sequence	Frequency				
	*In vitro*	*In vivo*			
	Pan 3^a^	Pan 1	Pan 2	Pan 3.1^b^	Pan 3.2^c^
WTLDRGY	5		1		2
PPNLKHS		2			
PNHSSRT		1			
PLVTAQN		1			
VPKQTQM		1			
LRGETVH		1			
PNWPHWP		1	1	1	
KFHLGAP		1	2		1
PPFGHTP			1		
HPNFLPA			1		
SPKTMKH			2		4
LLKDARL				4	
SNAGRNA				2	
LHNITST				1	
TPSTAFQ					1

a Only clones from the third round of *in vitro* panning were sequenced.

b 10 minute post-infusion BM harvest.

c 30 minute post-infusion BM harvest.

As a first approach to evaluating cell binding specificities of the peptides identified in our screen, murine BMCs were incubated with clonal stocks of biotinylated phage and cell binding was detected by flow cytometry. Compared to the other clones, the phage with the WTLDRGY sequence demonstrated the highest degree of binding to murine BMCs (**Fig. 1B**). In order to test the cell-binding specificity as a function of the order of residues in the WTLDRGY sequence, a control phage with the inverse sequence (YGRDLTW) was synthesized and binding to murine BMCs was evaluated by flow cytometry. Compared with the WTLDRGY sequence, the inverted control sequence demonstrated substantially reduced binding to murine BMCs (**Fig. 1C**).

We next sought to determine the specific residues within the WTLDRGY sequence that conferred cell-binding. To evaluate this, we developed a panel of seven scanning alanine mutant phage (one for each of the residues of the WTLDRGY sequence) and utilized our flow cytometry assay to assess phage binding to murine BMCs. Scanning alanine mutagenesis of the WTLDRGY sequence demonstrated that the sequence WTLXXGY is specifically mediating leukocyte binding (**Fig. 1D**).

**Figure 2:**
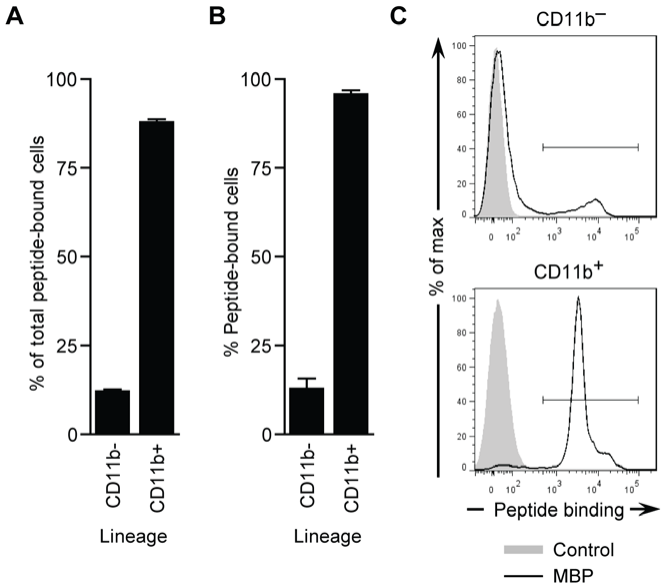
Assessment of MBP synthetic peptide binding to BMCs. (**A**–**C**) The heptapeptide sequence from the WTLDRGY phage clone was incorporated into a 14 aa synthetic peptide (CWTLDRGYCSAEKA) termed MBP, with a cysteine bridge, three C-terminal flanking residues derived from the phage pIII protein, and a biotinylated lysine residue at position 13, for flow cytometric analysis of binding to murine BMCs. (**A**) Percentage of total MBP-bound BMCs that are CD11b^+^ or CD11b^−^ by flow cytometry. (**B**) Percentage of CD11b^+^ (myeloid) and CD11b^−^ BMCs that are bound by MBP. (**C**) Representative histograms for data in **B**. Gray histograms indicate control staining. Data in **A** and **B** are means ± s.e.m. of *n*  =  3 experiments.

To better resolve the binding specificity of the WTLDRGY clone to individual murine BMC populations, a 14 amino acid (aa) version of the peptide (CWTLDRGYCSAEKA) was synthesized, including a cysteine bridge, three C-terminal flanking residues based on the phage pIII protein, and a biotinylated lysine residue at position 13. The resulting peptide labeled a significant percentage of total BMCs (data not shown). Greater than 87% of these cells expressed the myeloid lineage marker, CD11b (**Fig. 2A**), indicating the binding specificity is primarily restricted to the myeloid (CD11b^+^) lineage. Furthermore, the resulting peptide labeled greater than 95% of total CD11b^+^ BMCs compared to controls (**Fig. 2B,C**), and this binding was not specific to any particular CD11b^+^ cell type (i.e. CD11b^+^Ly6G^+^ or CD11b^+^Ly6G^−^ cells) (data not shown). Thereafter, we designated the WTLDRGY sequence: myeloid cell-binding peptide (MBP).

### Stable incorporation of MBP into Ad5

**Figure 3:**
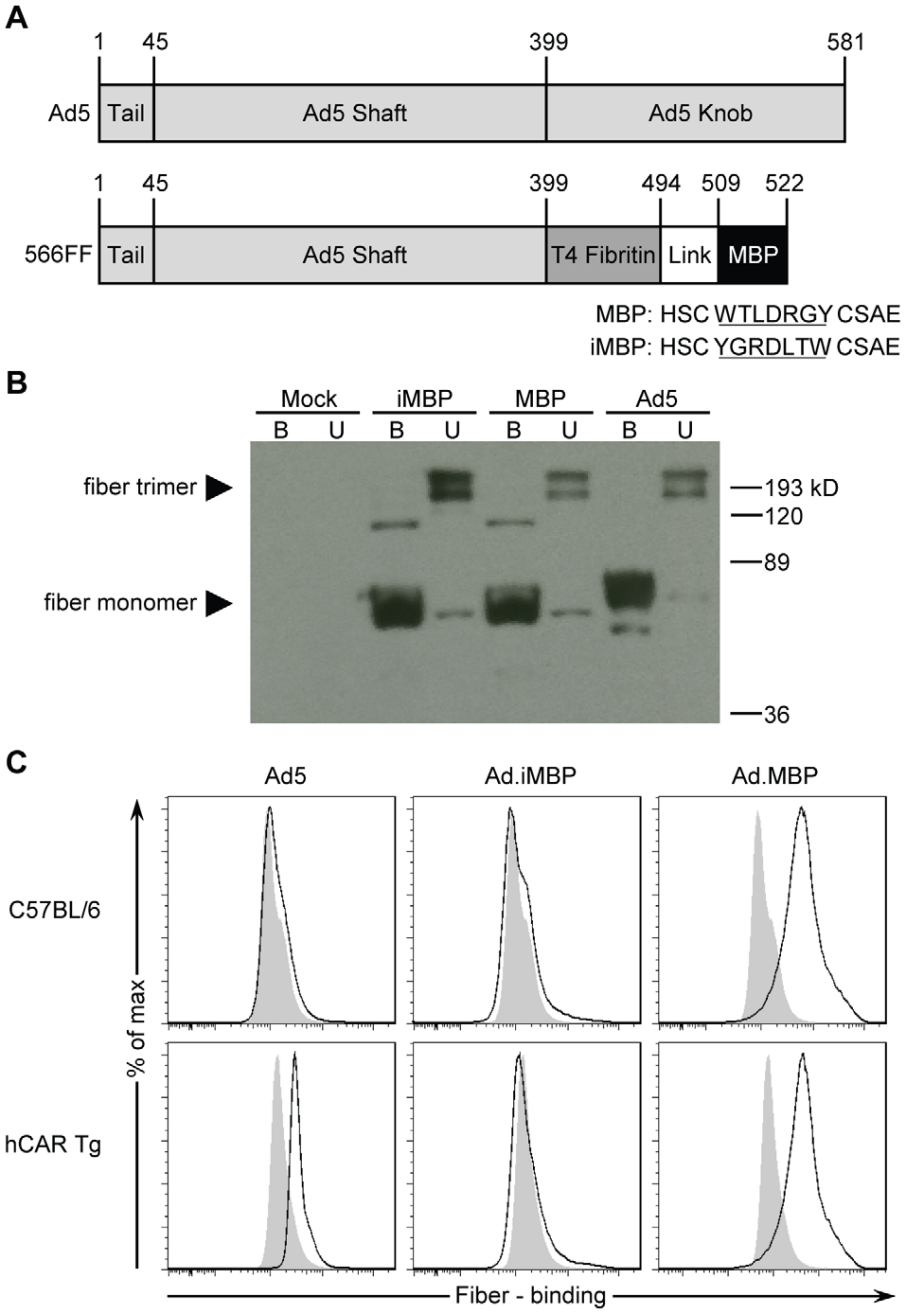
MBP fibers are viable and retain myeloid cell-binding specificity. (**A**) Diagram comparing wild type Ad5 and the MBP fibers. The MBP fiber is derived from the 566FF platform that our group has previously developed [20]. Briefly, the knob domain is removed and replaced with the trimerization region from the T4 phage fibritin protein fused to the MBP targeting ligand via a flexible linker ([GGGS]_4_). (**B**) Assessment of MBP fiber viability by Western blot. 293T cells were harvested 48 h after transfection with expression plasmids for wild type Ad5, MBP, or inverted MBP (iMBP) fibers. Protein supernatants from 293T cells were incubated at either 95°C (boiled, B) or room temperature (unboiled, U) prior to SDS-PAGE separation. Unboiled samples demonstrate that the majority of fibers are trimerized. (**C**) Evaluation of fiber binding to CD11b^+^ (myeloid) BMCs from wild type (C57BL/6) and transgenic mice that express hCAR (hCAR Tg). 293T protein supernatants from **B** were added to BMCs and fiber-bound CD11b^+^ cells were detected by flow cytometry. A representative experiment is shown for **C**.

Ad5 cellular entry is mediated by distinct binding and internalization events; the knob domain of Ad5 fiber initiates attachment through interactions with CAR [3], while internalization is mediated by distinct interactions between integrins and the RGD motif within the Ad5 penton [4]. Our group has previously developed a genetic Ad targeting platform, based on key fiber modifications. Specifically, the knob domain was deleted to ablate its broad tropism to CAR-expressing cells, and a 95 aa trimerization domain of the T4 phage fibritin protein was inserted to improve stability and allow display of novel targeting ligands (**Fig. 3A**) [19], [20]. The MBP sequence (WTLDRGY) as well as the inverse sequence YGRDLTW (termed inverted MBP [iMBP]) were first inserted into a vector expressing this fiber-fibritin (FF) platform (**Fig. 3A**) [9], [20], [28] to verify that genetic incorporation produced stable fibers that maintained trimerization potential (**Fig. 3B**). Following verification of viable fiber protein expression and trimerization, we next assessed whether recombinant FF-MBP retained myeloid cell-binding specificity (**Fig. 3C**). Notably, only FF-MBP, but not FF-iMBP or Ad5 fibers, demonstrated binding to murine CD11b^+^ BMCs (**Fig. 3C**), indicating that myeloid cell-binding specificity is entirely dependent on the presence of the MBP sequence. In contrast, detection of Ad5 fiber binding to CD11b^+^ cells was only detected when the BMCs were isolated from transgenic mice that express the native Ad5 receptor, hCAR [29] (**Fig. 3C**).

**Figure 4:**
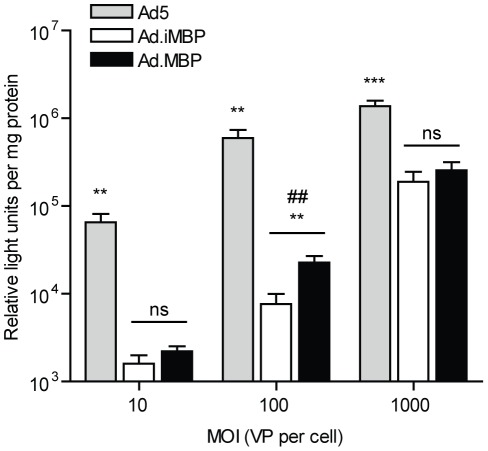
Knob-deleted Ad virus (Ad.MBP and Ad.iMBP) transduction of the CAR-expressing cell line, 293. Ad5.Luc, Ad.iMBP.Luc, and Ad.MBP.Luc were added to QBI-293A cells in 24-well plates at increasing multiplicities of infection (MOI) for 24 h at 37°C before luciferase activity was assessed at 24 h post-infection. Data are means ± s.d. of a representative experiment with triplicate samples. ##, *P*<0.01; **, *P*<0.01 compared to both Ad5 and Ad.iMBP; ***, *P*<0.001 compared to both Ad5 and Ad.iMBP.

Since fiber trimerization and myeloid cell-binding were maintained, the FF-MBP and FF-iMBP (as a control) sequences were then genetically incorporated into non-replicating Ad genomes by homologous recombination in *E. coli* and the resultant genomes for Ad.MBP and Ad.iMBP were used for viral rescue in E1-complementing cells. Traditionally, recombinant Ad5 is propagated and purified from the hCAR-expressing HEK-293 cell line; however, recombinant knob-deleted vectors (such as Ad.MBP and Ad.iMBP in the current study) are first rescued in a wild type Ad5 fiber-complementing cell line, termed 293-F28 [30]. After several rounds of amplification in 293-F28 helper cells, the resulting ‘mosaic’ virus (i.e. Ad5/MBP or Ad5/iMBP) was then added to HEK-293 cells for final amplification of the homogeneous, non-mosaic virions. MBP- and iMBP-FF incorporation were verified by Western blot on purified Ad particles (data not shown), and transduction of the HEK-293 cell line was assessed (**Fig. 4**) prior to subsequent functional characterization. As expected, the knob-deleted Ad vectors (Ad.MBP and Ad.iMBP) demonstrated significantly reduced (*P*<0.001) transduction of CAR-expressing non-myeloid cells, compared to Ad5 (**Fig. 4**).

### Functional characterization of Ad.MBP binding

**Figure 5:**
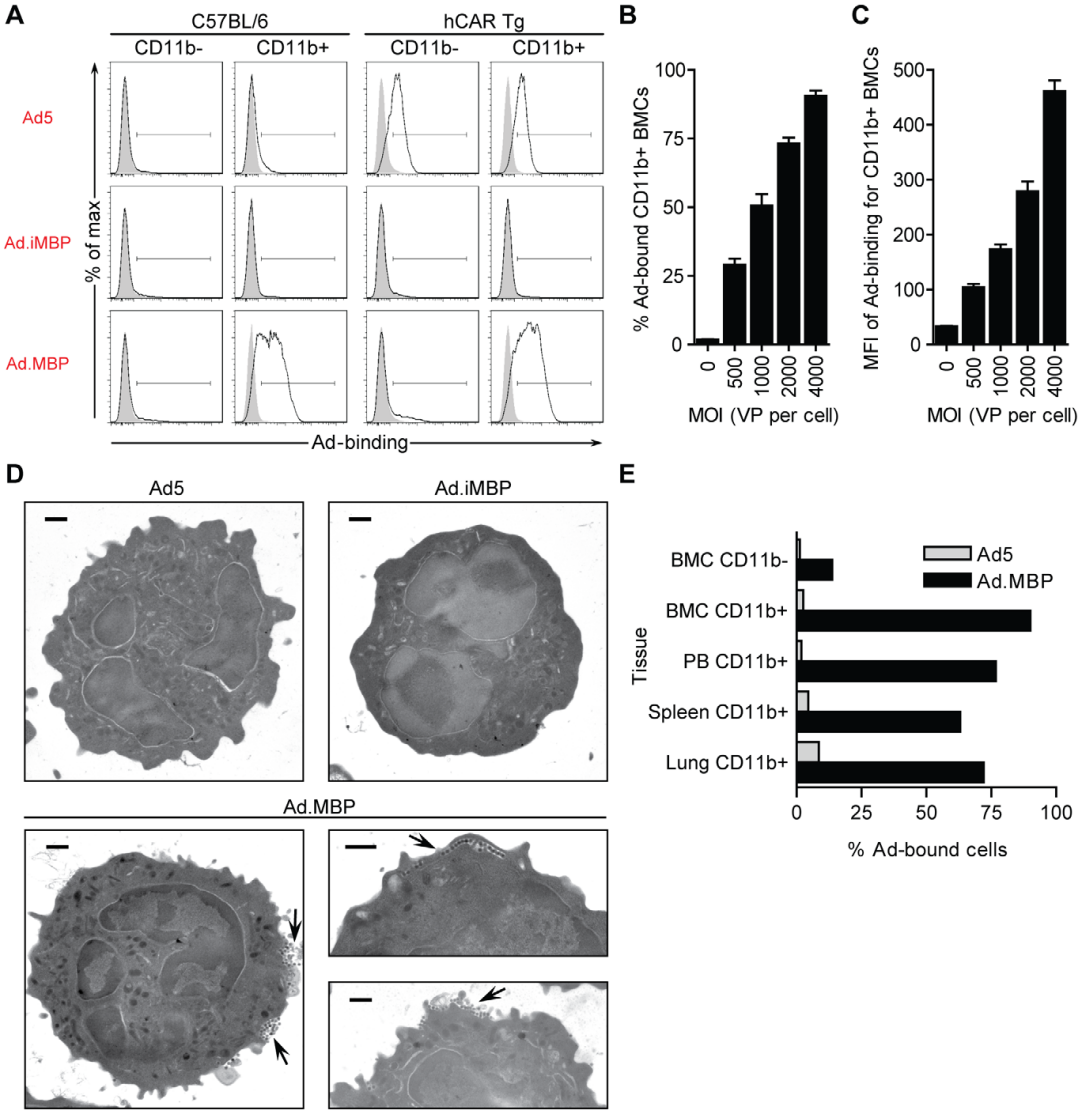
Genetic incorporation of MBP fiber into Ad5 (Ad.MBP) retains myeloid cell-binding specificity. (**A**) Assessment Ad5-, Ad.iMBP-, and Ad.MBP-binding (2,000 VP per cell) at 4°C to myeloid (CD11b^+^) and non-myeloid (CD11b^−^) BMCs from C57BL/6 and transgenic mice that express hCAR (hCAR Tg) by flow cytometry. (**B,C**) Evaluation of Ad.MBP-binding to CD11b^+^ BMCs at increasing MOI at 4°C. (**B**) Percentage of total CD11b^+^ BMCs bound by Ad.MBP. (**C**) Mean fluorescent intensity (MFI) of Ad.MBP-binding to CD11b^+^ BMCs. (**D**) Evaluation of Ad5-, Ad.iMBP-, and Ad.MBP-binding (500 VP per cell) at 4°C to murine BM neutrophils was also determined by transmission electron microscopy (TEM). Arrows indicate Ad.MBP virions along the cell surface. Scale bar, 500 nm. (**E**) Assessment of Ad5- or Ad.MBP-binding (2,000 VP per cell) at 4°C to CD11b^+^ cells from BM, peripheral blood (PB), spleen, and lung by flow cytometry. Binding to CD11b^−^ BMCs is also shown for comparison. Representative experiments are shown for **A**, **D**, and **E**. Data in **B** and **C** are means ± s.d. of a representative experiment with triplicate samples.

We next assessed whether Ad.MBP maintained specificity for the appropriate leukocyte subsets. To do so, Ad5, Ad.iMBP, and Ad.MBP were added to total murine BMCs (2,000 VP per cell) at 4°C, and cell-bound virions were detected by flow cytometric analysis. As with MBP fiber-binding, Ad.MBP virions demonstrated a high degree of binding to CD11b^+^ cells independent of hCAR expression, and did not show any appreciable binding to CD11b^−^ cell types (**Fig. 5A**). Ad5 binding was not detected on any BMC populations except those derived from transgenic mice that express hCAR (**Fig. 5A**). The Ad.iMBP control virus did not bind any BMC populations, including those from hCAR^+^ mice (**Fig. 5A**). Furthermore, Ad.MBP cell binding was dose dependent and was only saturating at levels greater than 4,000 VP per cell (**Fig. 5B,C**).

In addition to flow cytometric binding studies, we utilized transmission electron microscopy (TEM) to visualize Ad.MBP binding to BM neutrophils. The transmission electron micrographs demonstrate numerous virion filled ruffles along the surface of the neutrophil (**Fig. 5D**). In agreement with the flow cytometry data, neither Ad5 nor Ad.iMBP was detected on these populations by TEM (**Fig. 5D**). Taken together, these experiments (**Fig. 5A,D**) demonstrate our ability to retarget virus binding to myeloid cell populations. Furthermore, Ad.MBP, but not Ad5, bound to CD11b^+^ cells in the peripheral blood, spleen, and lung demonstrating that Ad.MBP has the capacity to bind myeloid (CD11b^+^) cell populations from a variety of tissues *ex vivo* (**Fig. 5E**).

### Functional characterization of Ad.MBP transduction

**Figure 6:**
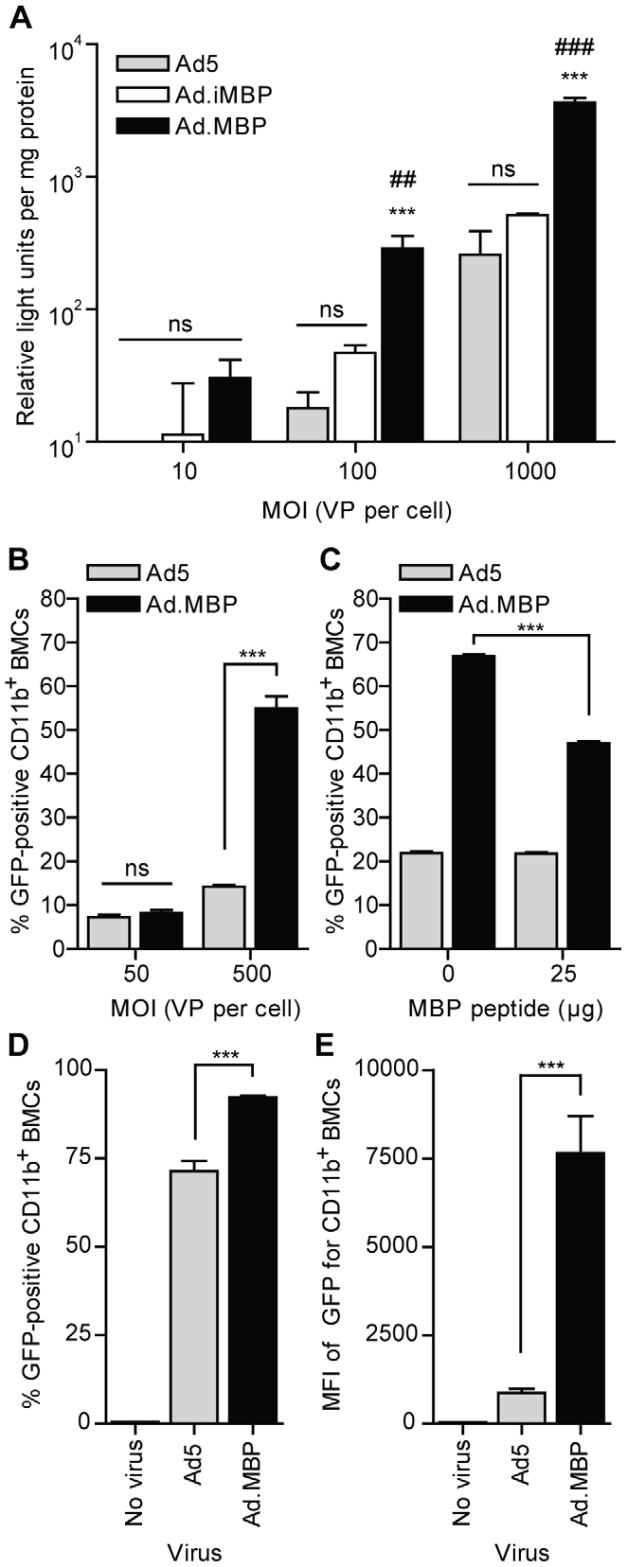
Myeloid cell-binding specificity of Ad.MBP enables enhanced transduction of CD11b^+^ BMCs *in vitro*. (**A**) Transduction of total BMCs by Ad5.Luc, Ad.iMBP.Luc, or Ad.MBP.Luc at increasing MOI as measured by luciferase activity (relative expression units) and normalized to protein concentration of individual samples. Total BMCs were mixed with virus at 4°C for 60 min, then washed, and plated at 37°C for 24 h prior to luciferase assay. ***, *P*<0.001 compared to Ad5; ##, *P*<0.01 compared to AdiMBP; ###, *P*<0.001 compared to AdiMBP. (**B**) Flow cytometric analysis of CD11b^+^ BMCs transduced with Ad5.GFP or Ad.MBP.GFP at increasing MOI. Total BMCs were mixed with virus at 4°C for 20 min, then washed, and plated at 37°C for 24 h. CD11b^−^ BMCs were not efficiently transduced by either virus (data not shown). ***, *P*<0.0001. (**C**) Flow cytometric analysis of CD11b^+^ BMCs pre-incubated with the 14 aa MBP peptide and then transduced with Ad5.GFP or Ad.MBP.GFP. Total BMCs from transgenic mice that express hCAR were mixed with the MBP peptide (25 µg) prior to virus (MOI  =  500) as in **B**, or mixed with virus alone (MOI  =  500), then washed, and plated at 37°C for 24 h. ***, *P*<0.0001. (**D**,**E**) Total BMCs were mixed with Ad5.GFP or Ad.MBP.GFP (MOI  =  500) at 37°C for 2 h, then washed, and plated at 37°C for a total of 24 h, before flow cytometric analysis was performed. (**D**) Percentage of total CD11b^+^ BMCs transduced by either virus. CD11b^−^ BMCs were not efficiently transduced by either virus (data not shown). (**E**) Mean fluorescent intensity (MFI) of GFP for CD11b^+^ BMCs. ***, *P*<0.001. Data in **A–E** are means ± s.d. of representative experiments with triplicate samples.

Finally, we assessed whether Ad.MBP binding to myeloid cells would translate to enhanced infectivity of these populations, compared to Ad5. To evaluate this, we first incubated total murine BMCs with Ad5.Luc, Ad.iMBP.Luc, or Ad.MBP.Luc at 4°C, and cultured the cells for 24 h before infectivity was assessed by luciferase assay. At higher MOIs, Ad.MBP.Luc demonstrated significantly higher transduction of total murine BMCs, compared to both Ad5.Luc and Ad.iMBP.Luc (*P*<0.001) (**Fig. 6A**). To determine if Ad.MBP transduction of BMCs was specific to the myeloid lineage, we incubated murine BMCs with Ad5.GFP or Ad.MBP.GFP as above, and the cells were cultured for 24 h before infectivity of specific cell types was assessed by flow cytometry. As expected, both Ad5 and Ad.MBP transduced less than 5% of CD11b^−^ cell types (data not shown). However, compared to Ad5, Ad.MBP significantly enhanced transduction of CD11b^+^ cells up to four-fold (*P*<0.0001) (**Fig. 6B**) and resulted in significantly higher GFP^+^ intensity of signal per cell (MFI) (data not shown). The myeloid cell-specificity of MBP was further demonstrated by pre-incubation of hCAR^+^ BMCs with the 14 aa MBP peptide, which significantly reduced Ad.MBP transduction of CD11b^+^ BMCs from mice, but not transduction with Ad5 (*P*<0.0001) (**Fig. 6C**). Furthermore, we assessed Ad.MBP transduction of CD11b^+^ cells under more physiological binding conditions. To do so, total murine BMCs were incubated with Ad5.GFP or Ad.MBP.GFP for 2 h at 37°C, after which unbound virus was removed and the cells were cultured for an additional 22 h (24 h total). Under these conditions, both viruses transduced CD11b^+^ cells; however, Ad.MBP transduced a significantly higher percentage of CD11b^+^ cells and resulted in a significantly higher GFP MFI than Ad5 (*P*<0.001) (**Fig. 6D,E**). Together, these experiments demonstrate that myeloid cell-binding (**Fig. 5**) via the MBP sequence (WTLDRGY) significantly enhances Ad.MBP transduction of target cells (**Fig. 6**).

## Discussion

Recent breakthroughs have highlighted the potential of gene therapy for a number of congenital and acquired illnesses. The critical factor underlying improved therapeutic outcome is typically the efficiency of gene delivery to diseased cells. Currently, virus-based vectors are the most efficient means of gene transfer to a number of cell types. Specifically, Ad5-based vectors represent an efficient gene delivery platform and are currently being evaluated for a number of clinical endpoints. However, as with most viral-based vectors, Ad5 vectors are still limited by a lack of specific and efficient gene targeting to clinically-relevant cell types.

Our group has previously described knob-deleted Ad fiber platforms that ablate hCAR binding and facilitate stable display of novel targeting elements [19], [20]. Using these genetically-modified vector platforms, we and others [21], [22] have demonstrated the ability to target novel cell types in a hCAR-independent manner through incorporation of specific ligands, however, demonstration of targeting clinically relevant receptors *in vivo* are limiting [24].

Selective binding is an important first-step in achieving specific transduction; however, this strategy relies on physical access between the virus and the target cells. Thus, for intravascular delivery to extravascular targets, a mechanism must be in place to deliver the virus to the sites of pathology. We sought to target Ad to myeloid cell types, as these cells actively circulate, traffic to—and participate in—many chronic inflammatory diseases. As a first step in achieving this endpoint we utilized phage display biopanning to identify peptides that bind to myeloid (CD11b^+^) cells. One particular phage sequence, WTLDRGY, identified in sequential rounds of enrichment, was found to efficiently bind to CD11b^+^ but not CD11b^−^ cells (namely, B and T cells) from a number of tissues. Site-specific scanning alanine mutagenesis demonstrated that the ordinate sequence WTLXXGY provides the binding specificity. This myeloid cell-binding peptide (MBP) sequence was then synthesized and used for further characterization of the cell type binding specificity. We next sought to determine whether the specificity of MBP was maintained when incorporated into our fiber-fibritin platform. Despite its small size, the specificity was maintained when expressed as recombinant fibers, and finally when the recombinant fibers were genetically incorporated into virions; demonstrating that this peptide can efficiently redirect the binding of knob-deleted Ad virions. Compared to Ad5 and non-specific peptide insertions, myeloid cell-binding was only detected with Ad.MBP in multi-lineage total bone marrow populations. The myeloid cell-binding specificity directly translated to significantly improved transduction of the myeloid cell types in total BM over Ad5, which was an increase of up to four-fold over Ad5. This enhancement was significantly inhibited by pre-incubation of target cells with the recombinant WTLDRGY peptide. Furthermore, Ad.MBP transduction was evaluated after allowing bone marrow leukocytes to first bind to virus at 37°C instead of 4°C. Incubation of total BMCs with Ad.MBP, resulted in significantly higher transduction of CD11b^+^ leukocytes, compared to Ad5, however the difference was not as striking as with binding done at 4°C. This result is not surprising, as we would expect that incubation of Ad5 virions with highly phagocytic CD11b^+^ leukocytes (e.g. neutrophils) in a closed system would result in increased non-specific uptake of the Ad5 particles. Despite the increased transdcution with Ad5 under these conditions, the unique ability of Ad.MBP to specifically bind to CD11b^+^ cells translated to a nine-fold increase in transgene expression, compared to Ad5. Although we selected BM leukocytes as our panning target population, we show that MBP also binds myeloid leukocytes from an array of tissues (bone marrow, peripheral blood, spleen, and lung), indicating specificity is maintained for myeloid cells and has potential for use in a number of inflammatory disorders.

Overall, these myeloid cell-targeting studies characterize the specificity of Ad.MBP targeting *ex vivo* and are the first step in demonstrating the potential of this virus for targeting clinically relevant cell types *in vivo*. This vector may be suitable for *ex vivo* gene transfer of myeloid cell types for reinfusion and subsequent trafficking of therapeutics to inflammatory sites *in vivo*. Alternatively, intravenous delivery may be possible if myeloid cell-binding *in vivo* occurs with high efficiency. In either case, we hypothesize the gene-modified leukocytes will traffic intravascularly, extravasate towards inflammatory signals, and this inside-out delivery approach may enhance therapeutic delivery to chronic sites of inflammation. Current studies are underway to identify the cognate receptor of MBP, and to determine whether systemic targeting of myeloid cell types with Ad.MBP will allow virus/therapeutic egress and trafficking to extravascular sites of inflammation.

## Materials and Methods

### Ethics statement for animal use

C57BL/6J mice (Jackson Laboratory, Bar Harbor, ME) between the ages of 6 to 10 weeks old were used in this study. Transgenic hCAR (hCAR Tg) mice were a gift from Dr. Sven Pettersson (Karolinska Institute, Stockholm, Sweden). These mice express a truncated form of human CAR (hCAR) under control of the human ubiquitin-C promoter, allowing hCAR expression in a variety of tissues [29]. All methods were approved by the Institutional Animal Care and Use Committee (IACUC) at the University of Alabama at Birmingham (Animal Protocol Number [APN] 7618 and 9313) and performed according to their and the National Institutes of Health guidelines.

### Cell lines

HEK-293 and 293T (Microbix, Toronto, Canada), QBI-293A (Qbiogene, Montreal, Canada), and 293-F28 cell lines were all propagated in Dulbecco's modified Eagle's medium (DMEM)-F12 medium supplemented with 10% (vol/vol) fetal calf serum (FCS), L-glutamine (2 mM), penicillin (100 I.U./mL) and streptomycin (100 µg/mL). The 293-F28 cell line, a derivative of HEK-293, expresses the Ad5 wild type fiber for mosaic virus propagation as described previously [30]. All cells were propagated at 37°C in a humidified atmosphere of 5% CO_2_.

### Phage display

For *in vivo* panning, 2×10^11^ plaque-forming units (pfu) of the Ph.D.-C7C cysteine-constrained heptapeptide M13 Phage Display Library (New England Biolabs, Ipswich, MA) was diluted in 200 µL α-MEM and infused into the tail vein (t.v.) of one mouse. After 10 min, BMCs were flushed from femoral and tibial marrow cavities with α-MEM supplemented with 2% FCS (α-MEM/2%). Cells were filtered through 40 µm nylon strainers and centrifuged at 250 *g*. Pellets were resuspended in erythrocyte lysis buffer (155 mM ammonium chloride, 20 mM sodium bicarbonate and 1 mM EDTA, pH 8), centrifuged at 250 *g*, and finally resuspended in phosphate buffered saline (PBS) supplemented with 2% FCS (FACS buffer). Cells of interest were isolated by fluorescence-activated cell sorting (FACS). Cell-bound phage were eluted with 0.2 M Glycine-HCl (pH 2.2), neutralized with 1 M Tris-HCl (pH 9.1), then amplified and sequenced according to NEB's instructions. This process was repeated two times with the amplified output phage after each round used as input for the subsequent panning experiment. For the third round of panning, two animals were each infused with 2×10^11^ pfu of the output phage from the second round of panning. One mouse was sacrificed after 10 min and the other mouse was sacrificed after 30 min. For *in vitro* panning, 2×10^11^ pfu of the same starting library from the manufacturer was added to 2.5×10^7^ BMCs isolated from naïve mice (as above) and incubated at 4°C for 6 h. Excess phage were removed by centrifuging the cells at 250 *g* and cells of interest were then isolated by FACS. Cell-bound phage were eluted, amplified and sequenced as described above for a total of three rounds of panning.

### Phage vector construction

M13-MBP phage mutants were synthesized by substituting an alanine for each of the seven residues of the MBP sequence (WTLDRGY). An inverse MBP (iMBP) control mutant with the MBP sequence in reverse (YGRDLTW) was also constructed. Ten different 56 basepair (bp) *Acc*65I-*Eag*I oligo linkers comprising the MBP, iMBP, or mutant MBP sequence were cloned into the *Acc*65I-*Eag*I-linearized M13KE cloning vector (New England Biolabs) for transformation of *E. coli* ER2738 without negative selection according to the New England Biolabs protocol. Phage DNA was isolated according to the New England Biolabs protocol and positive clones were identified by PCR and verified by sequencing.

### Phage preparation

Large scale stocks of M13 phage clones were prepared essentially according to the New England Biolabs protocol. Briefly, amplified phage were recovered by PEG precipitation of supernatants from 25 mL cultures and resuspended in 1 mL Tris-buffered saline (TBS) with 50% glycerol. Titers were determined by dilution plaque assays on *E. coli* ER2738. In order to detect phage binding to BMCs, the original (15 clones identified from phage panning) M13 phage preps were biotinylated with EZ-Link® Sulfo-NHS-LC-Biotin (Pierce, Rockford, IL) according to the manufacturer's protocol, while the M13-MBP mutant phage preps used in subsequent binding experiments were not biotinylated.

### Ad vector construction

The shuttle vectors, pKan-566FF-MBP and pKan-566FF-iMBP (used for Ad5 fiber replacement), were generated by cloning a 54 bp *Bam*HI-*Bcl*I oligo linker comprising the MBP (or iMBP) sequence into the *Bam*HI-linearized vector pKan-566FF-*Bae*I [9], [20]. The mammalian expression vectors, pVS-566FF-MBP and pVS-566FF-iMBP, containing the modified fiber genes, were then created by replacing the 1.5 kilobase (kb) *Age*I fragment from the expression vector pVS-566FF-ΔCd with similar sized *Age*I fragments from the above shuttle vectors. Plasmid pVS-566FF-ΔCd was constructed by deleting a 0.2 kb *Bam*HI-*Swa*I fragment, containing the Cd domain, from pVS-566FF-Cd [9], [20] and replacing it with a 58 bp oligo linker containing an *Age*I site. The expression vector pVSII containing the wild type Ad5 fiber gene is described elsewhere [28].

Recombinant non-replicating Ad genomes containing the modified MBP fiber genes (pAd.MBP.Luc, pAd.iMBP.Luc, and pAd.MBP.GFP) were then derived by homologous DNA recombination in *E. coli* BJ5183 between the *Eco*RI-digested shuttle vectors and *Swa*I-linearized, pVK700 and pVK900, as previously described [31]. The plasmids pVK700 and pVK900 are derivatives of pTG3602 [31] which contains full E1 and fiber gene deletions [32], [33]. In place of the E1 region, pVK700 and pVK900 express the firefly luciferase (Luc) and eGFP reporter genes, respectively, from the CMV immediate early (IE) promoter. Genome size was characterized by restriction digest and gel electrophoresis and sections of the genomes were sequenced to verify both the fiber and transgene regions.

### Viruses

Ad.MBP and Ad.iMBP viruses were first generated by transfection of 293-F28 cells with *Pac*I-digested pAd.MBP and pAd.iMBP genomes using Lipofectamine-2000 (Invitrogen, Carlsbad, CA) as described previously [30]. Rescued virions at this point are referred to as “mosaic” because they contain both the wild type Ad5 fibers and 566FF-MBP (or -iMBP) fibers. To generate virions with only 566FF-MBP or -iMBP fibers, we transduced QBI-293A cells with mosaic Ad5/MBP and Ad5/iMBP viruses following further rounds of amplification in 293-F28 cells. Cells were harvested after CPE was observed and virus was purified by two rounds of CsCl density ultracentrifugation and dialyzed in storage buffer (10% glycerol, 1 mM MgCl_2_, and 10 mM HEPES, pH 7.8). Isogenic control E1-deleted Ad5.Luc and Ad5.GFP vectors [19] (each expressing transgene from the CMV-IE promoter) were generated by infection of QBI-293A cells with purified virions. Cells were harvested after CPE was observed and virus was purified as above. Virus particle (VP) concentration was determined at 260 nm by using a conversion factor of 1.1×10^12^ VP per mL per absorbance unit as previously described [34].

### Recombinant fiber expression system

293T cells were seeded on 10-cm dishes prior to transfection with the expression plasmids pVS-566FF-MBP, pVS-566FF-iMBP, and pVSII (8.5 µg DNA per dish) using Lipofectamine-2000 (Invitrogen) according to the manufacturer's protocol. Cells (including mock transfected control) were harvested after 48 h, washed and then frozen in PBS (1 mL per 10-cm dish of cells). Protein supernatants (clarified of cell debris) were collected after three freeze/thaw cycles and protein concentrations were determined by *DC* Protein Assay (Bio-Rad, Hercules, CA).

### Western blotting

293T protein supernatants (10 µg total protein) or purified Ad5, Ad.iMBP, and Ad.MBP virions (1×10^9^ VP) were diluted in Laemmli sample buffer and incubated at either 95°C (boiled) or room temperature (unboiled) for 10 min and then separated by SDS-PAGE (10–12% resolving gels). Proteins were subsequently electroblotted onto PVDF membranes for western blot using the mouse 4D2 monoclonal antibody (mAb) to the Ad5 fiber tail domain (Lab Vision, Fremont, CA) followed by horseradish peroxidase-conjugated rabbit polyclonal secondary antibody to mouse IgG (Dako, Carpinteria, CA) as previously described [33]. Blots were developed using the Amersham ECL Plus Western Blotting Detection Kit (GE Healthcare, Piscataway, NJ). Precision Plus Protein Kaleidoscope Standards (Bio-Rad) were used to estimate molecular weights of bands.

### In vitro binding experiments

Prior to binding studies, BMCs (isolated as above), peripheral blood (isolated by cardiac puncture into heparinized tubes), and spleen cells (filtered through 40 µm strainers) were all centrifuged at 250 *g*. Lungs were removed, chopped into small pieces, resuspended in 1 mL Hank's Balanced Salt Solution (HBSS) supplemented with 0.14 Wünsch units Liberase (Roche, Indianapolis, IN) and 100 Units DNase I (Roche) and incubated for 25 min at 37°C while shaking [35]. Lung homogenates were then passed through 40 µm strainers and centriguged at 250 *g*. Cell pellets from all sources were resuspended in erythrocyte lysis buffer, centrifuged at 250 *g*, then washed and resuspended in FACS buffer, for subsequent experimentation. All binding experiments were carried out on ice or at 4°C. For all binding experiments, antibodies to various cell surface markers were included with secondary antibody staining to help distinguish various cell populations by flow cytometry as described below.

For phage and peptide binding, 5×10^8^ pfu biotinylated phage or 10 µg of biotinylated peptide were added to 1×10^6^ BMCs. The biotinylated peptide was synthesized at the Cleveland Clinic Lerner Research Institute Molecular Biotechnology Core (Cleveland, OH) and dissolved in PBS at 5 mg/mL. Cell-bound biotinylated phage or peptide was detected with APC-conjugated streptavidin (BD Pharmingen, San Diego, CA) by flow cytometry. Unbiotinylated phage-binding was detected with mouse monoclonal antibody to the M13 major coat protein pVIII (GE Healthcare) followed by PE-conjugated goat polyclonal secondary antibody to mouse IgG (SouthernBiotech, Birmingham, AL).

For fiber protein binding experiments, 575 µg (∼100 µL) of 293T protein supernatant was used to stain 1×10^6^ BMCs and cell-bound fiber was detected with mouse 4D2 mAb to the Ad5 fiber tail domain (Lab Vision, Fremont, CA) followed by Alexa Fluor 488 (A488)-conjugated goat polyclonal secondary antibody to mouse IgG (Invitrogen Molecular Probes, Eugene, OR).

For characterization of Ad-binding of BMCs, peripheral blood, spleen and lung, Ad.MBP, Ad.iMBP, or Ad5 were incubated with 1×10^6^ cells at different multiplicity of infection (MOI). Cellular-bound Ad was detected with rabbit polyclonal antiserum to Ad5 (as previously described [36]) followed by either A488-conjugated goat polyclonal or A647-conjugated chicken polyclonal secondary antibody to rabbit IgG (Invitrogen Molecular Probes).

### Transmission electron microscopy (TEM)

BMCs were isolated as above and then neutrophils were purified by negative magnetic bead-based antibody selection to remove T and B cells, erythrocytes, and monocytes/macrophages as described previously [37]. Ad5, Ad.iMBP, and Ad.MBP binding was carried out, as described above (MOI  =  500). Virus-bound cells were fixed in a modified Karnovsky's fixative (2% Paraformaldehye and 2% Glutaraldehyde in 0.1 M phosphate buffer). After fixation the specimens were rinsed several times with PBS followed by post-fixation with 1% osmium tetroxide in phosphate buffer for 1 h. After rinsing, the tissue specimens were dehydrated through a series of graded ethyl alcohols from 70 to 100%. After dehydration, the samples were embedded in EMbed 812 resin (Electron Microscopy Sciences, Hatfield, PA). Images were collected using a FEI Tecnai F20 FEG transmission electron microscope (FEI, Hillsboro, OR) at 80 KV in the UAB High Resolution Imaging Facility.

### In vitro transduction

For QBI-293A infection, Ad5.Luc, Ad.iMBP.Luc, or Ad.MBP.Luc at different MOI were incubated with 0.2×10^6^ QBI-293A cells in DMEM-F12 media as described above, and then luciferase activity was assayed at 24 h using the luciferase assay system (Promega, Madison, WI) and a TD-20/20 luminometer (Turner Designs, Sunnyvale, CA). Protein concentrations of cell culture samples were determined by *DC* Protein Assay (Bio-Rad) in order to normalize relative light units (RLU) values from different samples.

For BMC infection, BMCs were harvested and erythrocytes lysed as above. Ad5.Luc, Ad.iMBP.Luc, or Ad.MBP.Luc were incubated with 1×10^6^ BMCs at different MOI in α-MEM/2% for 60 min at 4°C. Ad.MBP.GFP or Ad5.GFP were incubated with 1×10^6^ BMCs at different MOI in α-MEM/2% for 20 min at 4°C or 2 h at 37°C. For both experiments, cells were washed twice to remove unbound virus and then plated in 1 mL of α-MEM supplemented with 20% FCS, SCF (50 ng/mL), IL-3 (20 ng/mL), and IL-6 (50 ng/mL) (cytokines from R&D Systems, Minneapolis, MN). For blocking experiments, 1×10^6^ BMCs from hCAR Tg mice were pre-incubated with 25 µg MBP peptide for 20 min at 4°C before addition of virus (MOI  =  500). After incubation for 24 h at 37°C, cells from all BMC experiments were harvested, and assayed for luciferase activity (as for QBI-293A experiment above) or stained for flow cytometry (and detection of GFP expression).

### Flow cytometry

Antibodies to CD11b (clone M1/70), Gr-1 (RB6-8C5), and Ly-6G (1A8), were all purchased from BD Pharmingen. Other antibodies were already mentioned. Antibody to CD16/32 (2.4G2; BD Pharmingen) was used to block endogenous mouse Fc Receptors before antibody staining. For binding studies, 2.4G2 was added after cells had been incubated with phage, peptide, 293T cell lysate, or virus but before staining with other antibodies. Data were acquired on a FACSCalibur or LSRII (BD Biosciences, Franklin Lakes, NJ) and analyzed with FlowJo v.7.6.1 (Tree Star, Inc., Ashland, OR).

### Statistical analysis

Data are presented as means ± s.d. or s.e.m. where appropriate. Statistical differences between groups were determined by analysis of variance (with Bonferroni post-test correction for comparisons of three or more groups) using Prism 4.0 (GraphPad Software, La Jolla, CA). Statistical significance was accepted at *P*<0.05.
